# Bis[μ-5-(2-pyrid­yl)tetra­zolato]-κ^3^ *N* ^1^,*N* ^5^:*N* ^2^;κ^3^ *N* ^2^:*N* ^1^,*N* ^5^-bis­[triaqua­zinc(II)] bis­(trifluoro­acetate) monohydrate

**DOI:** 10.1107/S1600536809025112

**Published:** 2009-07-04

**Authors:** Li Zhang

**Affiliations:** a Ordered Matter Science Research Center, College of Chemistry and Chemical Engineering, Southeast University, Nanjing 210096, People’s Republic of China

## Abstract

The title compound, [Zn_2_(C_6_H_4_N_5_)_2_(H_2_O)_6_](CF_3_CO_2_)_2_·H_2_O, was synthesized by hydro­thermal reaction of ZnBr_2_, CF_3_COOH and 2-(2*H*-tetra­zol-5-yl)pyridine. The Zn^II^ cation is coordinated by one N atom from the 5-(2-pyrid­yl)tetra­zolate anion, two N atoms from another 5-(2-pyrid­yl)tetra­zolate anion and three O atoms from three water mol­ecules in a distorted octa­hedral geometry. The tetra­zole ligands bridge the metal ions of the dimeric structure, and the dimers are located on crystallographic inversion centers. An inter­stitial solvent water mol­ecule is located on a crystallographic mirror plane, and the CF_3_COO^−^ counter-anions are also not coordinated to the metal complex. The F atoms of the anions are disordered with the F atoms statistically distributed over two positions in a 0.56 (3)/0.44 (3) ratio. All the water H atoms are involved in O—H⋯N and O—H⋯O hydrogen bonds with uncoordinated water O atoms, carboxyl­ate O atoms and tetra­zole N atoms. The inter­actions link the mol­ecules into a three-dimensional network.

## Related literature

For general background to metal-organic coordination compounds, see: Fu *et al.* (2007 ▶); Georgiev & MacGillivray (2007 ▶). For the crystal structures of related compounds, see: Zhao *et al.* (2008 ▶); Fu *et al.* (2008 ▶).
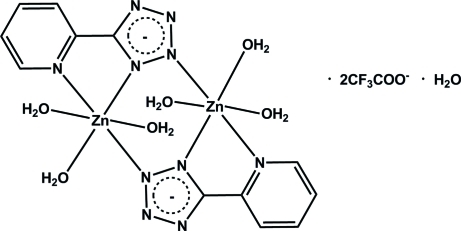


## Experimental

### 

#### Crystal data

[Zn_2_(C_6_H_4_N_5_)_2_(H_2_O)_6_](C_2_F_3_O_2_)_2_·H_2_O
*M* *_r_* = 775.18
Orthorhombic, 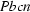

*a* = 9.1750 (18) Å
*b* = 14.722 (3) Å
*c* = 20.657 (4) Å
*V* = 2790.3 (10) Å^3^
*Z* = 4
Mo *K*α radiation
μ = 1.83 mm^−1^
*T* = 298 K
0.13 × 0.10 × 0.10 mm

#### Data collection

Rigaku Mercury2 diffractometer
Absorption correction: multi-scan (*CrystalClear*; Rigaku, 2005 ▶) *T*_min_ = 0.708, *T*_max_ = 0.833
27226 measured reflections
3197 independent reflections
2470 reflections with *I* > 2σ(*I*)
*R*_int_ = 0.074

#### Refinement

*R*[*F* ^2^ > 2σ(*F* ^2^)] = 0.041
*wR*(*F* ^2^) = 0.089
*S* = 1.12
3197 reflections
253 parameters
251 restraints
H atoms treated by a mixture of independent and constrained refinement
Δρ_max_ = 0.35 e Å^−3^
Δρ_min_ = −0.49 e Å^−3^

### 

Data collection: *CrystalClear* (Rigaku, 2005 ▶); cell refinement: *CrystalClear*; data reduction: *CrystalClear*; program(s) used to solve structure: *SHELXS97* (Sheldrick, 2008 ▶); program(s) used to refine structure: *SHELXL97* (Sheldrick, 2008 ▶); molecular graphics: *SHELXTL* (Sheldrick, 2008 ▶); software used to prepare material for publication: *SHELXTL*.

## Supplementary Material

Crystal structure: contains datablocks I, global. DOI: 10.1107/S1600536809025112/zl2226sup1.cif

Structure factors: contains datablocks I. DOI: 10.1107/S1600536809025112/zl2226Isup2.hkl

Additional supplementary materials:  crystallographic information; 3D view; checkCIF report

## Figures and Tables

**Table 1:** Hydrogen-bond geometry (Å, °)

*D*—H⋯*A*	*D*—H	H⋯*A*	*D*⋯*A*	*D*—H⋯*A*
O3—H3*WB*⋯O1*W*	0.809 (18)	2.03 (2)	2.788 (3)	156 (4)
O1—H1*WA*⋯O4	0.842 (18)	1.965 (19)	2.800 (3)	171 (4)
O3—H3*WA*⋯O5^i^	0.818 (18)	1.956 (19)	2.771 (3)	174 (4)
O2—H2*WB*⋯N3^ii^	0.825 (18)	2.08 (2)	2.856 (3)	156 (4)
O2—H2*WA*⋯O5^iii^	0.815 (18)	1.956 (19)	2.769 (3)	175 (4)
O1—H1*WB*⋯N2^ii^	0.818 (18)	2.02 (2)	2.821 (3)	165 (4)
O1*W*—H1*W*⋯O4^iv^	0.809 (18)	2.02 (2)	2.791 (3)	158 (4)

Symmetry codes: (i) 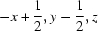; (ii) 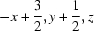; (iii) 
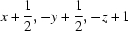
; (iv) 
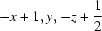
.

**Fig. 1.**
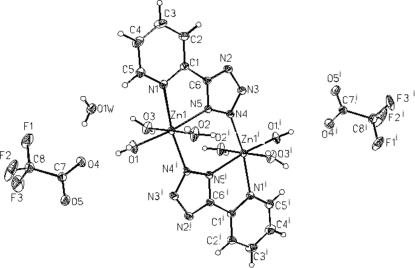
The molecular structure of the title compound, with the atomic numbering scheme. Displacement ellipsoids are drawn at the 30% probability level.

**Fig. 2.**
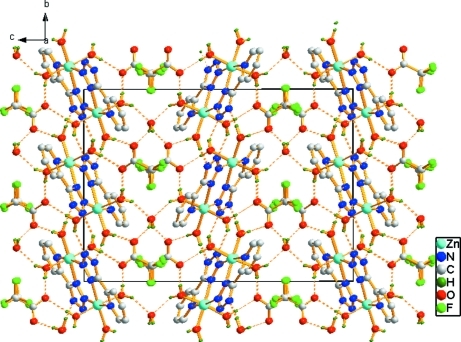
The crystal packing of the title compound, viewed along the a axis, showing the three dimensionnal hydrogen-bonding network (dashed lines). H atoms not involved in hydrogen bonding have been omitted for clarity.

## supplementary crystallographic information

### Comment

The construction of metal-organic coordination compounds has attracted much attention owing to their potential functions, such as permittivity, fluorescence and optical properties (Fu *et al.*, 2007; Georgiev *et al.*, 2007). Tetrazole compounds are a class of ligands excellently suited for the construction of such metal-organic coordination compounds because of the various coordination modes they exhibit towards metal ions (Zhao, *et al.* 2008; Fu *et al.*, 2008). We report here the crystal structure of one such metal-organic coordination compound, bis[(µ_2_-pyridinio-2-(2*H*-tetrazolato)- κ^3^N^1^,N^2^:N^5^)-hexa-aqua-di-zinc(II)]di-trifluoroacetate monohydrate.

In the title compound, each Zn^II^ cation is coordinated by one N atom from a 5-(2-pyridyl)tetrazolate anion, two *N,N*-chelating N atoms from another 5-(2-pyridyl)tetrazolate anion and three O atoms from three water molecules in a distorted octahedral geometry. The tetrazole groups act as µ_2_-bridges to link the Zn^II^ ions into dimers, which are located on crystallographic inversion centers. An interstitial solvate water molecule is located on a crystallographic mirror plane, and the CF_3_COO^-^ counter anions are also not coordinated to the metal complex. The F atoms of the anions are rotationally disordered with the F atoms statistically distributed over two positions with a 0.56 (3)/0.44 (3) ratio. The pyridine and tetrazole rings are nearly coplanar with each other and are twisted against each other by only 6.31 (1)°. The geometric parameters of the tetrazolate ring are comparable to those in related molecules (Zhao, *et al.* 2008; Fu *et al.*, 2008).

In the crystal structure, all the aqua H atoms are involved in O—H···N and O—H···O hydrogen bonds with the solvate aqua O (O1W), carboxyl O (O4, O5) and the tetrazole N (N2, N3) atoms. The interactions link the molecules into a three-dimensional network (Table 1 and Fig.2).

### Experimental

A mixture of 2-(2*H*-tetrazol-5-yl)pyridine (0.2 mmol), ZnBr_2_ (0.4 mmol), distilled water (1 ml) and CF_3_COOH (0.4 ml) was sealed in a glass tube and maintained at 323 K. Pure colorless block-shaped crystals suitable for X-ray analysis were obtained after 3 d, yield 55% based on ligand. Anal. Calcd. for C_16_ H_22_ F_6_ N_10_ O_11_ Zn_2_: C, 24.77%; H, 2.84%; N, 18.06%. Found: C, 24.69%; H, 2.79%; N, 17.98%. IR (KBr pellet, cm^-1^): 3421 (s), 3075 (w), 1726 (s), 1635 (s), 1610 (s), 1561 (w), 1475 (w), 1432 (w), 1367 (s), 1283 (s), 1257 (s), 1181 (w), 765 (w), 721 (w), 687 (w).

### Refinement

H atoms attached to C atoms were positioned geometrically and treated as riding, with C-H = 0.93 Å and with *U*_iso_(H) = 1.2*U*_eq_(C). H atoms of water molecules were located in difference Fourier maps and O—H distances were restrained to be 0.82 (2) Å with *U*_iso_(H) = 1.5*U*_eq_(O).

The F atoms of CF_3_COO^-^ anion are rotationally disordered over two positions. All C-F bonds were restrained to be the same within a standard deviation of 0.02 Å, and F···F distances within the disordered CF_3_ group were also restrained to be identical with the same standard deviation (PART and SADI command available in SHELXL-97 (Sheldrick, 2008)). The occupancy ratio for the two moieties refined to 0.56 (3) to 0.44 (3).

### Crystal data

**Table:**

[Zn_2_(C_6_H_4_N_5_)_2_(H_2_O)_6_](C_2_F_3_O_2_)_2_·H_2_O	*F*(000) = 1560
*M**_r_* = 775.18	*D*_x_ = 1.845 Mg m^−^^3^
Orthorhombic, *P**b**c**n*	Mo *K*α radiation, λ = 0.71073 Å
Hall symbol: -P 2n 2ab	Cell parameters from 2470 reflections
*a* = 9.1750 (18) Å	θ = 3.3–27.5°
*b* = 14.722 (3) Å	µ = 1.83 mm^−^^1^
*c* = 20.657 (4) Å	*T* = 298 K
*V* = 2790.3 (10) Å^3^	Block, colorless
*Z* = 4	0.13 × 0.10 × 0.10 mm

### Data collection

**Table:**

Rigaku Mercury2 diffractometer	3197 independent reflections
Radiation source: fine-focus sealed tube	2470 reflections with *I* > 2σ(*I*)
graphite	*R*_int_ = 0.074
Detector resolution: 13.6612 pixels mm^-1^	θ_max_ = 27.5°, θ_min_ = 3.3°
CCD profile fitting scans	*h* = −11→11
Absorption correction: multi-scan (*CrystalClear*; Rigaku, 2005)	*k* = −18→19
*T*_min_ = 0.708, *T*_max_ = 0.833	*l* = −26→26
27226 measured reflections	

### Refinement

**Table:**

Refinement on *F*^2^	Primary atom site location: structure-invariant direct methods
Least-squares matrix: full	Secondary atom site location: difference Fourier map
*R*[*F*^2^ > 2σ(*F*^2^)] = 0.041	Hydrogen site location: inferred from neighbouring sites
*wR*(*F*^2^) = 0.089	H atoms treated by a mixture of independent and constrained refinement
*S* = 1.12	*w* = 1/[σ^2^(*F*_o_^2^) + (0.0331*P*)^2^ + 2.0898*P*] where *P* = (*F*_o_^2^ + 2*F*_c_^2^)/3
3197 reflections	(Δ/σ)_max_ < 0.001
253 parameters	Δρ_max_ = 0.35 e Å^−^^3^
251 restraints	Δρ_min_ = −0.49 e Å^−^^3^

### Special details

**Table:**

Geometry. All esds (except the esd in the dihedral angle between two l.s. planes) are estimated using the full covariance matrix. The cell esds are taken into account individually in the estimation of esds in distances, angles and torsion angles; correlations between esds in cell parameters are only used when they are defined by crystal symmetry. An approximate (isotropic) treatment of cell esds is used for estimating esds involving l.s. planes.
Refinement. Refinement of F^2^ against ALL reflections. The weighted R-factor wR and goodness of fit S are based on F^2^, conventional R-factors R are based on F, with F set to zero for negative F^2^. The threshold expression of F^2^ > 2sigma(F^2^) is used only for calculating R-factors(gt) etc. and is not relevant to the choice of reflections for refinement. R-factors based on F^2^ are statistically about twice as large as those based on F, and R- factors based on ALL data will be even larger.

### Fractional atomic coordinates and isotropic or equivalent isotropic displacement parameters (Å^2^)

**Table:**

	*x*	*y*	*z*	*U*_iso_*/*U*_eq_	Occ. (<1)
Zn1	0.56081 (3)	0.12025 (2)	0.447600 (15)	0.02154 (11)	
N1	0.7732 (3)	0.12660 (15)	0.40077 (12)	0.0269 (5)	
N5	0.6601 (2)	−0.00968 (15)	0.47213 (11)	0.0227 (5)	
C1	0.8615 (3)	0.05501 (18)	0.41119 (13)	0.0242 (6)	
C3	1.0585 (4)	0.1271 (2)	0.35717 (17)	0.0431 (8)	
H3	1.1547	0.1278	0.3430	0.052*	
C2	1.0043 (3)	0.0530 (2)	0.39017 (15)	0.0328 (7)	
H2	1.0628	0.0026	0.3981	0.039*	
C4	0.9684 (4)	0.1997 (2)	0.34563 (17)	0.0439 (9)	
H4	1.0023	0.2500	0.3228	0.053*	
C5	0.8276 (4)	0.1973 (2)	0.36817 (16)	0.0359 (7)	
H5	0.7675	0.2470	0.3604	0.043*	
N2	0.8487 (3)	−0.10045 (16)	0.46066 (12)	0.0308 (6)	
C6	0.7937 (3)	−0.01880 (18)	0.44738 (13)	0.0232 (6)	
N3	0.7445 (3)	−0.14277 (16)	0.49503 (13)	0.0306 (6)	
N4	0.6325 (2)	−0.08873 (15)	0.50152 (11)	0.0234 (5)	
O1W	0.5000	0.1758 (2)	0.2500	0.0401 (8)	
H1W	0.563 (3)	0.212 (2)	0.241 (2)	0.060*	
O4	0.3097 (2)	0.29375 (15)	0.31366 (11)	0.0390 (5)	
O5	0.1974 (3)	0.41373 (15)	0.35629 (11)	0.0439 (6)	
C7	0.2553 (3)	0.3704 (2)	0.31174 (15)	0.0285 (6)	
O1	0.4894 (3)	0.24792 (13)	0.41823 (11)	0.0339 (5)	
H1WA	0.428 (3)	0.260 (2)	0.3893 (14)	0.051*	
H1WB	0.522 (4)	0.2972 (16)	0.4296 (18)	0.051*	
O2	0.6523 (3)	0.18073 (14)	0.52952 (11)	0.0394 (6)	
H2WA	0.670 (4)	0.151 (2)	0.5621 (13)	0.059*	
H2WB	0.697 (4)	0.2292 (18)	0.5291 (19)	0.059*	
O3	0.4655 (3)	0.07205 (15)	0.36187 (11)	0.0382 (6)	
H3WA	0.420 (4)	0.0246 (17)	0.3576 (18)	0.057*	
H3WB	0.490 (4)	0.089 (2)	0.3263 (12)	0.057*	
C8	0.2574 (4)	0.4177 (2)	0.24534 (16)	0.0383 (8)	
F1	0.3505 (12)	0.3818 (7)	0.2048 (5)	0.067 (2)	0.56 (3)
F2	0.286 (2)	0.5036 (5)	0.2486 (8)	0.099 (4)	0.56 (3)
F3	0.1294 (9)	0.4106 (11)	0.2175 (6)	0.101 (4)	0.56 (3)
F1'	0.3771 (14)	0.4040 (15)	0.2124 (8)	0.114 (5)	0.44 (3)
F2'	0.241 (2)	0.5056 (6)	0.2495 (9)	0.081 (4)	0.44 (3)
F3'	0.1517 (15)	0.3909 (10)	0.2077 (8)	0.081 (4)	0.44 (3)

### Atomic displacement parameters (Å^2^)

**Table:**

	*U*^11^	*U*^22^	*U*^33^	*U*^12^	*U*^13^	*U*^23^
Zn1	0.02240 (17)	0.01646 (16)	0.02576 (18)	−0.00013 (13)	0.00141 (14)	0.00342 (13)
N1	0.0262 (12)	0.0216 (12)	0.0329 (13)	−0.0012 (10)	0.0054 (10)	0.0065 (10)
N5	0.0239 (12)	0.0162 (11)	0.0281 (12)	0.0025 (9)	0.0040 (10)	0.0033 (9)
C1	0.0240 (15)	0.0228 (14)	0.0258 (15)	−0.0029 (12)	0.0039 (12)	−0.0014 (11)
C3	0.0308 (17)	0.046 (2)	0.052 (2)	−0.0089 (16)	0.0140 (16)	−0.0024 (16)
C2	0.0266 (15)	0.0314 (16)	0.0406 (19)	0.0002 (14)	0.0058 (14)	−0.0002 (14)
C4	0.041 (2)	0.0367 (18)	0.054 (2)	−0.0119 (15)	0.0144 (17)	0.0106 (16)
C5	0.0365 (17)	0.0273 (16)	0.0438 (19)	−0.0011 (14)	0.0071 (15)	0.0111 (14)
N2	0.0278 (13)	0.0239 (13)	0.0406 (15)	0.0073 (10)	0.0087 (11)	0.0063 (10)
C6	0.0227 (14)	0.0217 (13)	0.0251 (14)	0.0026 (11)	0.0024 (12)	−0.0013 (11)
N3	0.0323 (14)	0.0203 (12)	0.0394 (15)	0.0073 (11)	0.0085 (12)	0.0063 (11)
N4	0.0236 (12)	0.0166 (11)	0.0301 (13)	0.0023 (10)	0.0024 (10)	0.0026 (9)
O1W	0.054 (2)	0.0308 (18)	0.0356 (19)	0.000	0.0115 (17)	0.000
O4	0.0455 (14)	0.0298 (11)	0.0418 (13)	0.0104 (10)	0.0031 (11)	0.0040 (10)
O5	0.0642 (16)	0.0362 (12)	0.0314 (12)	0.0172 (12)	0.0148 (11)	0.0070 (10)
C7	0.0260 (15)	0.0286 (16)	0.0311 (15)	−0.0017 (12)	−0.0020 (13)	0.0027 (13)
O1	0.0426 (13)	0.0163 (10)	0.0428 (14)	−0.0028 (10)	−0.0170 (11)	0.0042 (9)
O2	0.0622 (16)	0.0264 (12)	0.0296 (12)	−0.0194 (11)	−0.0146 (12)	0.0060 (9)
O3	0.0556 (16)	0.0317 (12)	0.0272 (12)	−0.0181 (11)	−0.0036 (11)	−0.0010 (10)
C8	0.046 (2)	0.0387 (18)	0.0302 (18)	0.0042 (16)	0.0016 (15)	0.0037 (15)
F1	0.114 (6)	0.055 (4)	0.032 (3)	0.011 (3)	0.026 (3)	−0.003 (3)
F2	0.204 (11)	0.037 (4)	0.055 (6)	−0.035 (5)	0.039 (6)	0.006 (4)
F3	0.061 (4)	0.169 (10)	0.073 (6)	0.012 (5)	−0.030 (3)	0.057 (6)
F1'	0.072 (6)	0.177 (13)	0.093 (10)	0.055 (7)	0.056 (6)	0.088 (8)
F2'	0.175 (11)	0.033 (4)	0.035 (6)	0.015 (5)	−0.010 (7)	0.011 (4)
F3'	0.129 (8)	0.066 (5)	0.048 (5)	−0.017 (6)	−0.038 (6)	−0.018 (4)

### Geometric parameters (Å, °)

**Table:**

Zn1—O1	2.081 (2)	N2—N3	1.344 (3)
Zn1—O2	2.088 (2)	N3—N4	1.307 (3)
Zn1—O3	2.098 (2)	N4—Zn1^i^	2.113 (2)
Zn1—N4^i^	2.113 (2)	O1W—H1W	0.809 (18)
Zn1—N1	2.177 (2)	O4—C7	1.235 (3)
Zn1—N5	2.179 (2)	O5—C7	1.239 (4)
N1—C5	1.336 (4)	C7—C8	1.538 (4)
N1—C1	1.347 (3)	O1—H1WA	0.842 (18)
N5—C6	1.335 (3)	O1—H1WB	0.818 (18)
N5—N4	1.337 (3)	O2—H2WA	0.815 (18)
C1—C2	1.380 (4)	O2—H2WB	0.825 (18)
C1—C6	1.459 (4)	O3—H3WA	0.818 (18)
C3—C4	1.373 (5)	O3—H3WB	0.809 (18)
C3—C2	1.379 (4)	C8—F2	1.294 (8)
C3—H3	0.9300	C8—F3'	1.304 (9)
C2—H2	0.9300	C8—F2'	1.306 (9)
C4—C5	1.374 (4)	C8—F1'	1.307 (9)
C4—H4	0.9300	C8—F1	1.308 (7)
C5—H5	0.9300	C8—F3	1.312 (7)
N2—C6	1.332 (4)		
			
O1—Zn1—O2	88.72 (9)	N2—C6—N5	111.1 (2)
O1—Zn1—O3	85.89 (9)	N2—C6—C1	128.0 (2)
O2—Zn1—O3	174.52 (9)	N5—C6—C1	120.9 (2)
O1—Zn1—N4^i^	94.53 (9)	N4—N3—N2	109.4 (2)
O2—Zn1—N4^i^	91.59 (9)	N3—N4—N5	109.5 (2)
O3—Zn1—N4^i^	89.76 (9)	N3—N4—Zn1^i^	125.28 (17)
O1—Zn1—N1	96.52 (9)	N5—N4—Zn1^i^	125.18 (17)
O2—Zn1—N1	88.98 (10)	O4—C7—O5	128.3 (3)
O3—Zn1—N1	90.71 (10)	O4—C7—C8	115.9 (3)
N4^i^—Zn1—N1	168.94 (9)	O5—C7—C8	115.8 (3)
O1—Zn1—N5	173.03 (9)	Zn1—O1—H1WA	128 (3)
O2—Zn1—N5	91.03 (9)	Zn1—O1—H1WB	127 (3)
O3—Zn1—N5	94.22 (9)	H1WA—O1—H1WB	105 (4)
N4^i^—Zn1—N5	92.44 (8)	Zn1—O2—H2WA	121 (3)
N1—Zn1—N5	76.51 (8)	Zn1—O2—H2WB	124 (3)
C5—N1—C1	117.7 (2)	H2WA—O2—H2WB	112 (4)
C5—N1—Zn1	126.3 (2)	Zn1—O3—H3WA	126 (3)
C1—N1—Zn1	115.72 (17)	Zn1—O3—H3WB	123 (3)
C6—N5—N4	105.1 (2)	H3WA—O3—H3WB	108 (4)
C6—N5—Zn1	112.54 (17)	F2—C8—F3'	118.4 (11)
N4—N5—Zn1	142.25 (17)	F3'—C8—F2'	104.6 (8)
N1—C1—C2	122.5 (3)	F2—C8—F1'	90.5 (13)
N1—C1—C6	114.1 (2)	F3'—C8—F1'	105.5 (8)
C2—C1—C6	123.4 (3)	F2'—C8—F1'	106.5 (9)
C4—C3—C2	119.0 (3)	F2—C8—F1	107.3 (8)
C4—C3—H3	120.5	F3'—C8—F1	88.9 (9)
C2—C3—H3	120.5	F2'—C8—F1	121.2 (12)
C3—C2—C1	118.7 (3)	F2—C8—F3	106.3 (8)
C3—C2—H2	120.6	F2'—C8—F3	90.2 (11)
C1—C2—H2	120.6	F1'—C8—F3	120.8 (10)
C3—C4—C5	119.1 (3)	F1—C8—F3	105.8 (6)
C3—C4—H4	120.4	F2—C8—C7	113.5 (8)
C5—C4—H4	120.4	F3'—C8—C7	112.7 (9)
N1—C5—C4	122.8 (3)	F2'—C8—C7	112.8 (8)
N1—C5—H5	118.6	F1'—C8—C7	113.9 (8)
C4—C5—H5	118.6	F1—C8—C7	113.4 (6)
C6—N2—N3	104.9 (2)	F3—C8—C7	110.1 (6)

Symmetry codes: (i) −*x*+1, −*y*, −*z*+1.

### Hydrogen-bond geometry (Å, °)

**Table:**

*D*—H···*A*	*D*—H	H···*A*	*D*···*A*	*D*—H···*A*
O3—H3WB···O1W	0.81 (2)	2.03 (2)	2.788 (3)	156 (4)
O1—H1WA···O4	0.84 (2)	1.97 (2)	2.800 (3)	171 (4)
O3—H3WA···O5^ii^	0.82 (2)	1.96 (2)	2.771 (3)	174 (4)
O2—H2WB···N3^iii^	0.83 (2)	2.08 (2)	2.856 (3)	156 (4)
O2—H2WA···O5^iv^	0.82 (2)	1.96 (2)	2.769 (3)	175 (4)
O1—H1WB···N2^iii^	0.82 (2)	2.02 (2)	2.821 (3)	165 (4)
O1W—H1W···O4^v^	0.81 (2)	2.02 (2)	2.791 (3)	158 (4)

Symmetry codes: (ii) −*x*+1/2, *y*−1/2, *z*; (iii) −*x*+3/2, *y*+1/2, *z*; (iv) *x*+1/2, −*y*+1/2, −*z*+1; (v) −*x*+1, *y*, −*z*+1/2.
